# Protocol for genomic editing in human resting primary NK cells and NK-92 cells via CRISPR-Cas9 ribonucleoproteins

**DOI:** 10.1016/j.xpro.2024.103123

**Published:** 2024-06-26

**Authors:** Tias Verhezen, Ho Wa Lau, Jonas Van Audenaerde, An Wouters, Evelien Smits, Jorrit De Waele

**Affiliations:** 1 Center for Oncological Research, Integrated Precision and Personalized Oncology Network (IPPON), University of Antwerp, Antwerp, Belgium

**Keywords:** cancer, immunology, CRISPR

## Abstract

Here, we present a protocol to perform CRISPR-Cas9 genome editing in human resting primary natural killer (NK) and NK-92 cells. We describe steps for guide RNA selection, ribonucleoprotein (RNP) complex formation, delivery via Nucleofection, and analysis of CRISPR edits to assess editing efficiencies. This protocol offers a tool for functional studies in NK cells, paving the way for potential applications in immunotherapy and beyond. We also discuss limitations such as off-target effects and cell-type-specific considerations.

## Highlights

• Steps for isolating peripheral blood mononuclear cells from healthy donor buffy coats
• Magnetic purification of human resting primary NK cells
• CRISPR-Cas9 RNP editing of resting primary NK cells and NK-92 cells via Nucleofection
• Editing efficiency analysis via Sanger sequencing (gene) and flow cytometry (protein)

## Abstract (editor highlights)

Publisher’s note: Undertaking any experimental protocol requires adherence to local institutional guidelines for laboratory safety and ethics.

## Graphical abstract

**Figure:**
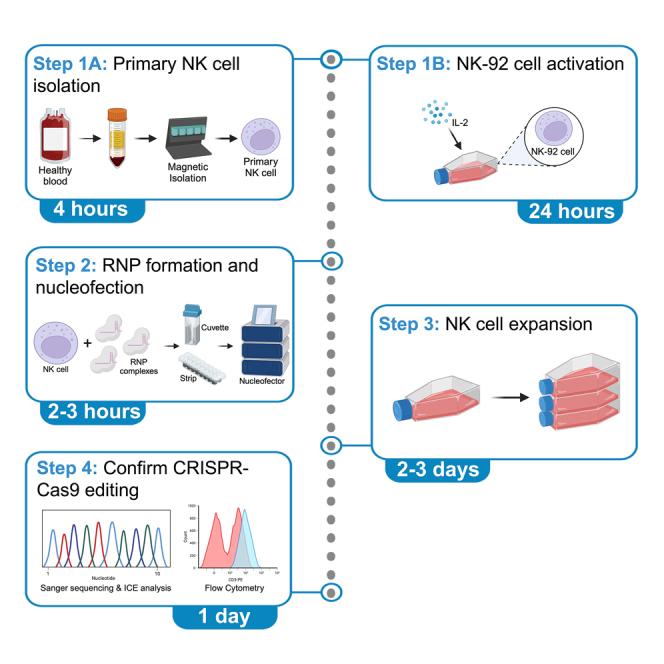

## Before you begin

The CRISPR-Cas9 system has revolutionized the field of molecular biology as a versatile genome-editing tool with a broad range of applications, including exciting possibilities for medical advancements in the field of cellular therapies. Guided by a short RNA molecule, Cas9 is targeted to a genomic locus and creates a DNA double-strand break. When the targeted locus is repaired by the non-homologous end joining (NHEJ) pathway, this error-prone repair mechanism re-ligates the double-strand breaks, introducing small insertions or deletions (indels) at the breakpoint. Indels within a coding exon of the gene can lead to frameshift mutations and premature stop codons, resulting in a knockout of the gene. This protocol represents a reliable and reproducible CRISPR-Cas9 method for precise genome editing in resting primary NK cells and NK-92 cells.

Genetic engineering of human NK cells via stable delivery systems of Cas9, such as viral vectors, presents important constraints. Lentiviral and retroviral transduction, for instance, induces NK cell apoptosis and yields only a restricted number of edited cells.^1^^,^^2^^,^^3^ Concurrently, the sustained expression of Cas9 increases the potential for off-target genomic modifications.^4^ Considering these limitations, we have chosen to employ ribonucleoprotein (RNP) complex delivery via Nucleofection. RNP complexes consist of (multiple) synthetic guide (sg)RNA(s) and preformed Cas9 protein. Unlike the traditional crRNA:tracrRNA system, sgRNA requires no annealing step, as it is already a single piece RNA molecule that performs well for CRISPR-Cas9 applications. The retention of the Cas9 enzyme within the cells for a span of approximately 24 h provides an adequate time frame for effective cellular edits while minimizing off-target interactions.

Our protocol employs a novel multi-guide approach with three distinct sgRNAs for efficient gene knockout in primary NK cells and the NK-92 cell line. The simultaneous use of multiple sgRNAs targeting the same genomic region facilitates the generation of multiple double-strand breaks at the intended genomic target locus, thereby improving the likelihood of successful gene knockout. By combining the delivery of pre-formed RNP complexes with a multi-sgRNA approach, gene editing is very efficient and precise.

The optimization process preceding this protocol considered both the concentration and the ratio of sgRNA and Cas9 enzyme to form the RNP complexes. These optimized ratios were not described in literature before. Additionally, we have optimized the Nucleofection programs for the Lonza 4D Nucleofector device for both resting primary human NK cells and NK-92 cells. Researchers can choose between Nucleofection cuvettes or strips based on the desired cell quantity for editing, providing flexibility and adaptability to experimental needs.

In the first section, we will go over the steps prior to starting the CRISPR-Cas9 protocol for primary human NK and NK-92 cells. These steps involve selecting the sgRNAs and designing PCR primers to amplify the specific gene region that will be edited. Utilizing software such as CLC Workbench (Qiagen) or SnapGene (Dotmatics) to digitally annotate your target gene, sgRNAs, and PCR primers can enhance the visualization of this process.

### Institutional permissions

All activities involving human blood specimens must adhere to the applicable ethical guidelines set by institutional and governmental authorities. Explicit consent should be acquired from participants prior to utilizing human blood for experimental purposes. In the present study, adherence to ethical standards was ensured through approval from the local Ethics Committee at Antwerp University Hospital-University of Antwerp (Antwerp, Belgium) under project ID 5488. The selection of blood donors and the blood collection process were conducted in accordance with the laws of Belgium and the policies established by the Belgian Red Cross. This process involves donors consenting to the use of their blood for purposes beyond transfusions, such as experimental research.

When carrying out this protocol, it is essential to follow Biosafety Level 2 (BSL2) procedures, utilize appropriate equipment, and work within facilities designed for this level of biosafety. Specifically, the handling of NK-92 cells in genetic engineering projects requires BSL-2 containment. Additionally, BSL-2 containment is required when working with human blood, blood components, and primary NK cells.

Application of CRISPR-Cas9 technology is considered genetic engineering, resulting in genetically modified organisms (GMOs). Genetic engineering is generally regulated by national laws and guidelines. The researcher should make him-/herself aware of these rules to ensure adherence.

### sgRNA selection

**Timing: 1 h**

Here, we explain how to select sgRNA(s) for your gene of interest. This protocol has been optimized for a multi-guide strategy to enhance knockout scores through the synergy among three different guides (1b.). Still, we also provide information on the single-guide strategy option (1a.).

1. Select and order sgRNA(s) for your gene of interest.
  ***Note:*** It is advisable to choose sgRNAs situated in the earliest exons to increase the likelihood of achieving a functional knockout (KO).
  a. When opting for a single-guide strategy, launch the Synthego CRISPR design tool (https://design.synthego.com/#/).
    i. Fill in your genome (homo sapiens) and gene of interest.
    ii. Pick the 3–5 highest scoring guide RNAs (high on-target activity and low off-target potential) to order.
      ***Note:*** The sgRNAs will be summarized in the ‘recommended guides’ tab. We recommend including several guides to screen during the optimization process.
  b. When using the multi-guide strategy, you can order the Gene Knockout Kit to test the combination of several guides (https://www.synthego.com/products/crispr-kits/gene-knockout-kit).
    i. Fill in your genome (homo sapiens) and gene of interest to order the kit.
      ***Note:*** The Synthego CRISPR Design Tool is optimized for individual guide editing (one guide introduced at a time). The GKOv2 Kits, on the other hand, use a multi-guide strategy. The guides included in the multi-guide combination are designed to work in synergy.
      ***Note:*** You will receive the information on the guides after the order, which you will need to design PCR primers.
      ***Note:*** If you cannot find your protein/gene of interest right away, check homologous names for your gene of interest. The Hugo Gene Nomenclature Committee's www.genenames.org can be consulted for this.
      ***Note:*** If you are opting for the multi-guide strategy, you can order the sgRNAs separately from the Synthego website for follow-up experiments instead of re-ordering the kit (https://www.synthego.com/order/crispr-kits/synthetic-sgrna/).
      ***Alternative:*** Other CRISPR design web tools include http://crispr.mit.edu and ‘Benchling’. In addition, guides can be sourced from published (whole-genome) CRISPR screens.

### PCR primer design and optimization of PCR conditions

**Timing: 1–2 h**

Here, we explain how to design forward and reverse PCR Primers for ICE (Inference of CRISPR Edits) Analysis to calculate the editing efficiencies of your CRISPR-Cas9 gene editing. It is recommended to design at least two primer pairs per gene of interest or per set of guide RNA’s. By testing multiple pairs, you have a better chance of finding one that provides efficient and specific amplification of your target region. Primer design software tools such as Primer3 and primer-BLAST may assist in your design and checking off-target amplification.

2. Select around 500 bp of genomic DNA on each side of the guide sequence, using an annotation tool as mentioned earlier. For multi-guide samples, select around 500 bp of genomic DNA on each side the furthest upstream and downstream guides.
3. Design the forward and reverse primers at least 150 bp upstream/downstream from the guide RNA cut site to allow for optimal sequencing quality. If using multi-guide, design the forward and reverse primers at least 150 bp from the outer sgRNA cut sites.

**CRITICAL:** Design primers with amplicon lengths between 400–800 bp (with an optimal length of 500 bp), a melting temperature of 60°C (range: 55°C–65°C), that are 18–22 bp in length and have 45%–60% GC content.

4. Order your custom DNA oligos via your manufacturer of choice, such as Integrated DNA Technologies.
5. Upon arrival of your PCR primers, optimize the PCR for your targeted region.
  a. Perform a screen with different annealing temperatures per primer pair.
  b. Confirm successful PCR reactions by visualizing results using Gel Electrophoresis.

***Note:*** Bands should be clearly visible and without a smear, which is indicative for off-target amplification.

## Key resources table

**Table:** 

REAGENT or RESOURCE	SOURCE	IDENTIFIER
**Biological samples**		

Healthy donor blood	Flemish Red Cross	N/A

**Chemicals, peptides, and recombinant proteins**		

Alt-R S.p. Cas9 nuclease	Integrated DNA Technologies	Cat# 1081058
Synthetic guide RNAs	Synthego	
Lymphoprep density gradient medium	STEMCELL Technologies	Cat# 07851
DNase-1	Roche	Cat# 10104159001
Protector RNase inhibitor	Merck	Cat# 3335399001
QuickExtract DNA extraction solution	Lucigen	Cat# QE0905T
Ethylenediaminetetraacetic acid (EDTA)-Na_2_ salt	Merck	Cat# 1084180250
Bovine serum albumin (BSA)	Sigma-Aldrich	Cat# A1662-1L
Human recombinant interleukin 2 protein (rhIL-2)	ImmunoTools	Cat# 11340025
Human recombinant interleukin 15 protein (rhIL-15)	ImmunoTools	Cat# 11340155
Fetal bovine serum (FBS)	Thermo Fisher Scientific	Cat# 10270106
Horse serum	Thermo Fisher Scientific	Cat# 16050122
L-glutamine (200 mM)	Thermo Fisher Scientific	Cat# 25030-024
Penicillin-streptomycin (10,000 U/mL)	Thermo Fisher Scientific	Cat# 15140122
RPMI 1640 medium, HEPES	Thermo Fisher Scientific	Cat# 52400025
BD FACSFlow sheath fluid	BD Biosciences	Cat# 342003
MEM α, GlutaMAX supplement, no nucleosides	Thermo Fisher Scientific	Cat# 32561037
Sodium azide (NaN_3_)	Merck	Cat# 769320
Human serum, normal	Sigma-Aldrich	Cat# S1-100ML

**Critical commercial assays**		

Gene knockout kit v2	Synthego	
MojoSort human NK cell isolation kit	BioLegend	Cat# 480054
P3 primary cell 4D-Nucleofector kit S (32 RXN) (Nucleofection strips)	Lonza	Cat# V4XP-3032
P3 primary cell 4D-Nucleofector Kit L (24 RXN) (Nucleofection cuvettes)	Lonza	Cat# V4XP-3024
Qubit dsDNA HS assay kit-500 assays	Thermo Fisher Scientific	Cat# Q32854
KAPA HiFi HotStart PCR kit, with dNTPs (250 U)	Roche	Cat# 7958897001
Wizard SV gel and PCR clean-up system	Promega	Cat# A9281
eBioscience Foxp3 / transcription factor staining buffer set	Invitrogen	Cat# 00-5523-00

**Experimental models: Cell lines**		

NK-92	DSMZ	DSMZ no.: ACC 488

**Oligonucleotides**		

PCR primers	Integrated DNA Technologies	

**Software and algorithms**		

ICE analysis	Synthego	https://ice.synthego.com/
CRISPR design tool	Synthego	https://design.synthego.com/#/
FlowJo v.10.8.0	FlowJo LLC	https://www.flowjo.com/

**Other**		

CELLSTAR 15 mL Falcon tubes	Greiner Bio-One	Cat# 188271
CELLSTAR 50 mL Falcon tubes	Greiner Bio-One	Cat# 227261
ABX Micros ES60 hematology analyzer	Horiba	
LS columns	Miltenyi Biotec	Cat# 130-042-401
MACS MultiStand (magnetic stand)	Miltenyi Biotec	Cat# 130-042-303
QuadroMACS separator	Miltenyi Biotec	Cat# 130-091-051
4D-Nucleofector core unit	Lonza	Cat# AWA-3001-B
4D-Nucleofector core unit, FL1	Lonza	Cat# AAF-1002B
4D-Nucleofector X unit	Lonza	Cat# AWA-3001-X
4D-Nucleofector X unit, FL1	Lonza	Cat# AAF-1002X
Qubit 4 quant starter kit wifi box	Thermo Fisher Scientific	Cat# Q33239
NovoCyte Quanteon flow cytometer system	Agilent	N/A
TC20 automated cell counter	Bio-Rad	Cat# 1450102

## Materials and equipment

Media should be kept at 4°C, then warmed to 37°C for use. They should also be kept sterile.

**Table:** NK-92 cell medium

Reagent	Final concentration	Amount
MEM α, GlutaMAX Supplement, no nucleosides	N/A	500 mL
Horse serum	12.5%	85 mL
Fetal Bovine serum	12.5%	85 mL
L-Glutamine	2 mM L-glutamine	6.7 mL
Penicillin/Streptomycin	1%	6.7 mL
**Total**	**N/A**	**683.4 mL**

**Table:** Primary NK cell and PBMC medium

Reagent	Final concentration	Amount
RPMI 1640	N/A	500 mL
Fetal Bovine serum	10%	50 mL
Sodium Pyruvate	1 mM	5 mL
L-Glutamine	2 mM L-glutamine	5 mL
Penicillin/Streptomycin	1%	5 mL
**Total**	**N/A**	**561.5 mL**

**Table:** EDTA-buffer

Reagent	Final concentration	Amount
ddH_2_O	N/A	1 L
EDTA-Na2 salt	100 mM	37.224 *g*
**Total**	**N/A**	**1 L**

Sterilize via filtration or autoclaving, keep sterile, store at 4°C for up to 12 months.

**CRITICAL:** EDTA causes skin and severe eye irritation. Always wear gloves, goggles, and lab coat while handling it.

**Table:** PBS-EDTA buffer

Reagent	Final concentration	Amount
PBS (1×)	N/A	990 mL
EDTA buffer (100 mM)	1 mM	10 mL
**Total**	**N/A**	**1 L**

Sterilize via filtration or autoclaving, keep sterile, store at 4°C for up to 3 months.

**Table:** MACS buffer

Reagent	Final concentration	Amount
PBS-EDTA	N/A	983.25 mL
Bovine serum albumin (30%)	10 nM	16.75 mL
**Total**	**N/A**	**1 L**

Sterilize via filtration or autoclaving, keep sterile, store at 4°C for up to 3 months.

**Table:** Red blood cell lysis buffer (10×) stock solution

Reagent	Final concentration	Amount
ddH_2_O	N/A	1 L
Ammonium chloride (NH_4_Cl)	1.55 M	82.9 *g*
Potassium bicarbonate (KHCO_3_)	0.1 M	10 *g*
EDTA-Na_2_ salt	1 mM	370 mg
**Total**	**N/A**	**1 L**

Sterilize via filtration or autoclaving, keep sterile, store at 4°C for up to 12 months.

**CRITICAL:** NH_4_Cl is toxic by oral ingestion.

**Table:** Red blood cell lysis buffer (1×) working solution

Reagent	Final concentration	Amount
ddH_2_O	N/A	45 mL
Red blood cell lysis buffer (10×) stock solution	1×	5 mL
**Total**	**N/A**	**50 mL**

Sterilize via filtration or autoclaving, keep sterile, store at 4°C for up to 2 weeks.

**Table:** FACS buffer

Reagent	Final concentration	Amount
BD FACSFlow	N/A	946.7 mL
Bovine serum albumin (30%)	0.1%	3.3 mL
Sodium azide (NaN_3_; 1%)	0.05%	50 mL
**Total**	**N/A**	**1 L**

Store at 4°C for up to 6 months.

**Table:** RNP complex master mix

Reagent	Amount for cuvette (μL)	Amount for strip (μL)
P3 solution	89.4	22.35
Alt-R S.p. Cas9 Nuclease (62 μM)	2.4	0.6
sgRNA mix (100 μM)	7.2	1.8
Protector RNase inhibitor	1	0.25
Total volume	100	25

Make the complex in sterile and RNase free conditions. Keep at 19°C–22°C for up to 1 h for immediate use, at 4°C for up to one week, or at −20°C for up to 1 month.

**Table:** PCR reaction master mix

Reagent	Amount per reaction
DNA Polymerase (1 U/μL)	0.5 μL
Primer 1 (forward) (10 μM)	0.75 μL
Primer 2 (reverse) (10 μM)	0.75 μL
High fidelity buffer (5X)	5 μL
ddH_2_O	Up to 25 μL

Make fresh right before PCR amplification.

## Step-by-step method details

### Peripheral blood mononuclear cells (PBMC) isolation from buffy coats

**Timing: 2 h**

To initiate the editing of human innate immune cell populations *ex vivo*, we begin with PBMC isolation from healthy donor buffy coats obtained from the blood bank, followed by negative selection of NK cells. While we detail our preferred isolation method below, alternative methods or blood sources can also be employed.

**CRITICAL:** Work sterile to avoid contamination of the cells.

1. Disinfect the blood bag and tube. Cut the tube to release the blood in a 50 mL falcon tube (∼40 mL blood).
2. Fill two 50 mL tubes with 15 mL density gradient medium such as Ficoll-Paque or LymphoPrep.
3. Carefully layer the peripheral blood on top of the density gradient medium. Avoid mixing the blood and density gradient layers. Evenly divide the blood over the two tubes.
4. Centrifuge the tubes for 10 min at 890 × *g* with maximal acceleration speed. Inactivate the rotor brakes to effectively prevent mixing of the different phases obtained after centrifugation.

***Note:*** After density gradient centrifugation of blood, three distinct layers emerge. At the top is the plasma/serum, followed by the buffy coat containing platelets and white blood cells. The bottom layer consists mainly of red blood cells.

5. Collect the buffy coat layer from both tubes and transfer to two separate 50 mL falcon tubes. Add 40 mL PBS-EDTA and resuspend pellets.
6. Centrifuge 10 min at 450 × *g*. Remove supernatant containing platelets.
7. Resuspend pellet one in 25 mL red blood cell lysis buffer (1x working solution) and transfer the suspension to the second tube. Resuspend pellet two as well. Now you are left with one tube containing all PBMCs.
8. Add 50 U/mL DNase-1 to prevent clotting. Softly rotate tube for 1 min.
9. Centrifuge 5 min at 450 × *g*. Remove supernatant with lysed red blood cells.
10. Finally resuspend cells in 50 mL PBS-EDTA.
11. Filter cells using a cell strainer (70 μm) to prevent clotting and clogging of the columns in later steps.
12. Count PBMCs with an automated cell counter. Include trypan blue staining for viability assessment.

***Note:*** A viability of 90%–99% is expected after PBMC isolation.

**Pause point:** PBMCs can be kept overnight at 1–1.6 × 10^7^ cells/mL in supplemented RPMI medium in a 37°C incubator to start NK cell isolation the day after. Add 50 U/mL DNase-1 to prevent clotting.

### Primary NK cell isolation

**Timing: 2 h**

Here, we explain how NK cells can be isolated from PBMCs using NK cell Biotin-Antibodies, magnetic Streptavidin Nanobeads and isolation columns of choice. Cells can be isolated through negative selection, where the cells of interest are not bound by antibody-conjugated magnetic nanobeads. Negative selection is warranted to maintain resting state of NK cells during isolation. The magnetically labeled cells that remain in the column are not eluted in this protocol. Here we use BioLegend’s MoJo Sort.

13. Decide on the number of columns based on PMBCs available and amount of NK cells needed for downstream assays. One column can be loaded with 100 × 10^6^ PBMCs maximally.

***Note:*** Healthy donor blood contains 5%–20% NK cells.

**Figure 1:**
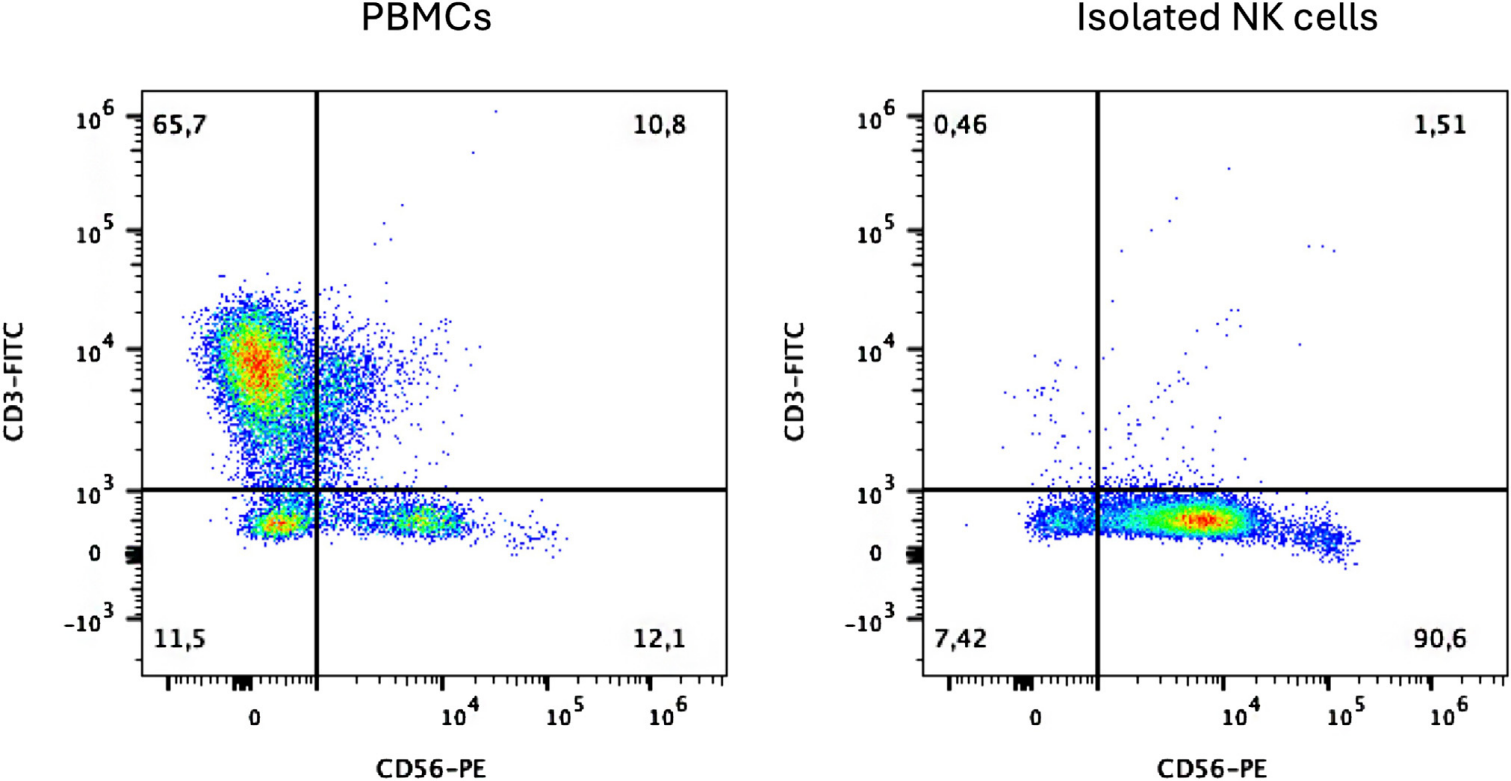
Representative flow cytometric plots of the percentage of CD3-CD56+ cells in freshly isolated PBMC (left) and after immunomagnetic enrichment (right) Gates were set on viable cells gating on the forward scatter (FSC) and side scatter (SSC) pseudocolor dot plots. CD3- CD56+ cells are identified as NK cells. A purity of more than 90% can be expected after immunomagnetic isolation.

***Note:*** A purity of more than 90% can be expected after immunomagnetic isolation.

14. Transfer the appropriate amount of PBMCs to a 50 mL tube.
15. Centrifuge at 300 × *g* for 5 min, then remove the supernatant. Resuspend in 100 μL MACS buffer per 10^7^ cells using a micropipette.
16. Use 10 μL of NK Cell Biotin-Antibody Cocktail per 10^7^ total cells. First, dilute the antibody cocktail 1/4 in MACS buffer.
17. Mix the diluted antibody with the cells and incubate on ice for 15 min.
18. Add 4 mL MACS and centrifuge at 300 × *g* for 5 min, then remove supernatant.
19. Resuspend pellet again in 100 μL MACS buffer per 10^7^ cells using a micropipette.
20. Use 10 μL of NK Cell Streptavidin NanoBeads per 10^7^ total cells. First, dilute the Nanobeads in 1/4 MACS buffer.
21. Mix the diluted Nanobeads with the cells and incubate on ice for 15 min.
22. Add 4 mL MACS and centrifuge at 300 × *g* for 5 min, then remove supernatant.
23. Finally resuspend the pellet in 3 mL MACS per column. The cells are ready to be placed on the columns.
24. Place labeled 15 mL falcon tubes underneath each column to collect the fraction containing the unlabeled NK cells.
25. Prewash columns with 3 mL MACS buffer.
26. Load 3 mL cell suspension per column, avoiding air bubbles and preventing column dryness. Wash each column 2–3 times with 3 mL MACS buffer.
27. Isolation is complete after 2–3 washes.
28. Centrifuge the isolated NK cells and remove the supernatant. Resuspend the pellets in primary NK cell medium and pool together.
29. Determine the purity of the isolated primary NK cells by staining CD3 and CD56 with fluorochrome-labeled antibodies for flow cytometry measurement. In Figure 1, a representative purity measurement is shown.
  a. Add 100,000 PBMC and 100,000 NK cells to two different polystyrene FACS tubes.
  b. Wash the samples by adding 2 mL of FACS buffer. Centrifuge cells at 450 × *g* for 5 min at 19°C–22°C. Discard the supernatant.
  c. Resuspend the cell pellets in 100 μL of FACS buffer and stain the cell pellets with 5 μL anti-CD3 FITC and 5 μL anti-CD56 PE antibodies.
  d. Incubate 15 min at room temperature (19°C–22°C) in the dark.
  e. Wash the samples by adding 2 mL of FACS buffer. Centrifuge cells at 450 × *g* for 5 min at 19°C–22°C. Discard the supernatant.
  f. Resuspend cell pellets in 100 μL FACS buffer.
  g. Acquire the stained cells on a flow cytometer and determine the percentage of CD3-CD56+ NK cells (representative plots are shown in Figure 1).

30. Count cells and leave overnight or proceed with CRISPR-editing.

***Note:*** To ensure optimal viability of resting primary NK cells after Nucleofection, we recommend using the NK cells directly after isolation.

***Note:*** Depending on your research question, primary human NK cells can be activated with cytokines prior to CRISPR editing. While this is expected to enhance efficacy and viability, our transfection protocol for resting human primary NK cells cannot be guaranteed as the most optimal method. In case of stimulated primary NK cells, pulse code optimization might be required. Refer to problem 1 for further details.

### NK-92 cell culture

**Timing: 24 h**

If you wish to work with the NK-92 cell line, you can find more information on culturing conditions and activation in this section. For enhanced editing efficiencies, we activate the NK-92 for 24 h, the day before Nucleofection.

31. Culture NK-92 cells in supplemented α-MEM medium (12.5% fetal bovine serum, 12.5% horse serum, 2 mM L-glutamine and 1% penicillin-streptomycin) at 0.25–0.4 × 10^6^ cells per mL.

**CRITICAL:** Include 100 U/mL recombinant human (rh) interleukin (IL)-2, as the proliferation and survival of NK-92 cells are IL-2 dependent.

32. Passage every 3–4 days.
33. 24 h prior to Nucleofection, culture NK-92 cells at a concentration of 1 × 10^6^ cells per mL. For activation stimulus, add the same amount of IL-2 per mL as for culturing purposes.

### Formation of RNP complexes

**Timing: 30 min**

In this section, we explain how to form the RNP complexes consisting of Cas9 enzyme (IDT) and sgRNA(s) (Synthego) in Nucleofection buffer named P3 solution (Lonza). The P3 kit from Lonza is the best choice for transfecting resting human primary NK cells and NK-92 cells among the 4D-Nucleofector kits they offer. The P3 Nucleofection buffer is composed of P3 supplement and P3 solution, requiring combination prior to use. To enhance the shelf-life of the P3 buffer, it can be made fresh prior to each experiment (see note for more information). We provide volume specifications for both Nucleofection strips and cuvettes to make RNP complexes. To save time, the complexes can be made beforehand (see pause point).

**CRITICAL:** Prepare the RNP complexes under RNase-free conditions. Use filter tips when handling genetic material.

34. Dissolve the sgRNA in TE buffer, which is provided by Synthego, by pulse vortexing for 30 s. Make a stock solution of 100 μM.

***Note:*** it is recommended to keep the resolved sgRNA in 6 μL aliquots in the freezer. To maintain their integrity, freeze-thaw cycles must be prevented.

35. Prepare the P3 Nucleofection buffer by adding the P3 supplement to the Nucleofector Solution. The ratio of Lonza P3 Nucleofector supplement to solution is 1–4.5.
  a. When working with strips, prepare 25 μL P3 buffer per condition (4.5 μL P3 supplement + 20.5 μL P3 solution).
  b. When working with cuvettes, prepare 100 μL buffer per condition (18 μL P3 supplement + 82 μL P3 solution).

***Note:*** After supplementation, the Nucleofector P3 solution retains its effectiveness for an additional three months when stored in the refrigerator. By preparing the needed amount of P3 solution fresh, you can enhance the shelf-life of your P3 products. The P3 supplement can also be added completely to the buffer after arrival instead of making fresh batches for each experiment.

36. Form the CRISPR-Cas9 RNP complex by mixing Cas9 enzyme, the sgRNAs and RNase inhibitor in the P3 solution according to Table 1.

**Table 1:** Optimized CRISPR-Cas9 RNP complex master mix

Reagent	Amount for cuvette (μL)	Amount for strip (μL)
P3 solution	89.4	22.35
Alt-R S.p. Cas9 Nuclease (62 μM)	2.4	0.6
sgRNA mix (100 μM)	7.2	1.8
Protector RNAse inhibitor	1	0.25
Total volume	100	25

***Note:*** When employing two or three distinct guide RNAs, ensure equal quantities of each individual guide by adhering to the specified volumes. For instance, in a cuvette setting with three different guides, add 2.4 μL of each guide, totaling 7.2 μL.

37. Incubate the RNP complexes for 10 min at 19°C–22°C.

**Pause point:** You can form the RNP complexes prior to the protocol. The complexes can be kept at 19°C–22°C for up to 1 h for immediate use, at 4°C for up to one week, or at −20°C for up to 1 month.

### Cell preparation

**Timing: 15 min**

NK cells need to be counted and washed before Nucleofection. Determine the number of cells you will edit, based on your downstream assays. Upon Nucleofection, 30%–50% of your cells can be lost. If you aim to only check editing efficiencies during optimization, you can start with 1–2 × 10^6^ cells (strip). If you aim to perform downstream assays, we recommend using at least 3–5 × 10^6^ NK cells (cuvette).

38. Count the freshly isolated primary NK cells or NK-92 cells that were activated overnight.
39. Transfer the right number of NK cells to an RNase-free and sterile Eppendorf tube. Use 1–2 × 10^6^ cells for strips and 3–5 × 10^6^ NK cells for cuvettes.
40. Centrifuge the cells (5 min, 200 × *g*) and remove the culture media.
41. Wash the cells by adding 1 mL PBS.
42. Centrifuge again (5 min, 200 × *g*) and remove PBS.

***Note:*** By washing the cells with PBS before Nucleofection, you help remove any residual RNases present in the medium. This step is crucial for ensuring the integrity and stability of the sgRNAs during the Nucleofection process.

***Note:*** Counting and washing of the NK cells can be performed during the 10 min incubation of the RNP complexes.

### Nucleofection

**Timing: 30 min**

In the process of introducing RNP complexes into NK cells, we employ Nucleofection with the Lonza 4D Nucleofector. Here, we delineate the optimized Nucleofection programs we have established and offer explicit instructions on using this Nucleofector device.

43. Prepare the primary NK cell or NK-92 culture medium by warming it up (needed in step 46).
44. Increase efficiency and save time by pre-programming the Nucleofector device (Lonza). Pre-select the well/cuvette positions you will use. Ensure to specify the buffer that is used to resuspend the cells before choosing the Nucleofection program.
  a. Use program CA-138 for primary human resting NK cells.
  b. Use program CM-137 for NK-92 cells.
45. Resuspend the washed and pelleted NK cells in the P3 solution containing the RNP complexes. Pipet carefully but thoroughly. Do not make bubbles.

***Note:*** Cells should not be resuspended in P3 solution for longer than 15 min. It is recommended to perform Nucleofection in multiple batches if you have many samples, depending on the speed of your pipetting work.

46. Immediately transfer the cell/RNP complex mixture to the Nucleofector strip or cuvette.
47. Gently tap the strip/cuvette. Make sure the mixture is uniformly distributed at the bottom of the strip/cuvette and that no bubbles are trapped between the electrodes.

***Note:*** Bubbles trapped between the electrodes can also be removed using a 20 μL pipette tip by pushing them aside.

48. Place the Nucleofector strip or cuvette in the Nucleofector device. Press ‘start’.
49. Take out the Nucleofector strip or cuvette and transfer back to a sterile environment.
50. Add pre-warmed culture medium (supplemented alpha-MEM for NK-92 cells; supplemented RPMI for primary NK cells) in each well immediately after Nucleofection without resuspending the cells. Do not remove the cells from the strip/cuvette yet.
  a. Add 1 mL in a cuvette.
  b. Add 125 μL to strips.
51. Let the cells recover in an incubator (37°C, 5% CO_2_) for 15 min.
52. Transfer the cells to plates or flasks.
  a. Culture the primary NK cells at 1 × 10^6^ cells/mL in a T25 bottle, 6-well plate, or 24-well plate, depending on the number of CRISPR-edited cells.
    ***Note:*** When keeping the primary NK cells in culture for longer periods of time, addition of low amounts of cytokines are needed to ensure proliferation and survival (i.e., IL-2 (100 U/mL) and IL-15 (5 ng/mL), added every three to four days).
  b. Culture the NK-92 cells at 0.5 × 10^6^ cells/mL in a T25 bottle, 6-well plate, or 24-well plate, depending on the number of CRISPR-edited cells.
    i. Add 100 U/mL IL-2 to the NK-92 cells.

### Analysis of editing efficiency on genomic level via sanger sequencing

**Timing: 2–3 days**

The following steps are applicable for both primary and NK-92 cells.

To quantify the editing efficiency and KO scores of the CRISPR reactions, Sanger sequencing of the targeted area is performed, followed by ICE analysis (Synthego’s free online tool).

53. Count and split the CRISPR-Cas9 edited NK cells after 2–3 days.
54. Pellet 0.5 × 10^6^ cells for the genetical analysis of editing efficiencies (see below).

**Pause point:** Keep dry cell pellets in the −80°C freezer up to six months before DNA extraction in step 55.

**CRITICAL:** Remember to include non-edited genomic DNA to use as a control for sequencing.

55. Resolve the pellet in 50 μL QuickExtract (QE) DNA Extraction Solution. Pulse vortex for 30 s. Transfer the resolved pellet to PCR tubes.

***Note:*** Using too much or too little QE Extraction Solution will interfere with your PCR results. See problem 2 for possible solutions.

56. Place the PCR tubes in the PCR thermocycler. Select the following program, according to Table 2.

**Table 2:** PCR cycling conditions

Steps	Temperature	Time	Cycles
Heating step 1	65°C	15 min	1
Heating step 2	98°C	10 min	1
Hold	4°C	Forever
57. Determine the genomic DNA concentration using a Nanodrop or Qubit. Use the QE DNA Extraction Solution as a blank for the Nanodrop measurement.
58. Prepare the PCR reaction master mix according to Table 3. Do not add genetic material to the master mix.

**Table 3:** PCR reaction master mix

Reagent	Amount per reaction
DNA Polymerase (1 U/μL)	0.5 μL
Primer 1 (forward) (10 μM)	0.75 μL
Primer 2 (reverse) (10 μM)	0.75 μL
High fidelity buffer (5X)	5 μL
ddH_2_O	Up to 25 μL
59. Distribute 20–24 μL of the PCR master mix to each PCR tube, depending on the amount of genomic DNA that will be added in the next step.
60. Add the same amount of genomic DNA of each condition to a separate PCR tube. Use 10–100 ng in a maximal volume of 5 μL.
  a. The total of the PCR reaction should be 25 μL.
61. Use following PCR cycling conditions (Table 4).

**Table 4:** PCR cycling conditions

Steps	Temperature	Time	Cycles
Initial Denaturation	95°C	3 min	1
Denaturation	98°C	20 s	25–35
Annealing	60°C–75°C (see Note)	15 s	
Extension	72°C	15 s	
Final extension	72°C	1 min	1
Hold	4°C	forever

***Note:*** The annealing temperature will be specific for each primer pair. The optimal annealing temperature for a specific primer set is likely to be different (higher) than when used in a conventional PCR buffer. An annealing temperature gradient PCR is recommended to determine the optimal annealing temperature with KAPA HiFi HotStart.

***Note:*** The cycling conditions in Table 4 were determined for the KAPA HiFi DNA Polymerase kit from Roche. Your polymerase and conditions of interest can also be used.

62. Confirm successful PCR reaction by visualizing results via Gel Electrophoresis before performing Sanger sequencing.
63. Measure DNA concentration using Qubit/Nanodrop to make sure the concentration is within range for Sanger sequencing.

***Note:*** See problem 2 for more information if DNA concentrations are too low.

64. Purify the PCR DNA with your method of choice. Here, the Promega Wizard SV PCR Clean-Up kit was used according to the protocol of the manufacturer.
65. Use your provider of interest to Sanger sequence the amplified DNA.

***Note:*** Ideally, sequence each control/edited sample with the forward and reverse primers, separately. Some edited cell populations may exhibit heterogeneity in the types and sizes of indels. By using both forward and reverse primers, you can capture a more comprehensive view of the variations present in the edited cell population.

**Figure 2:**
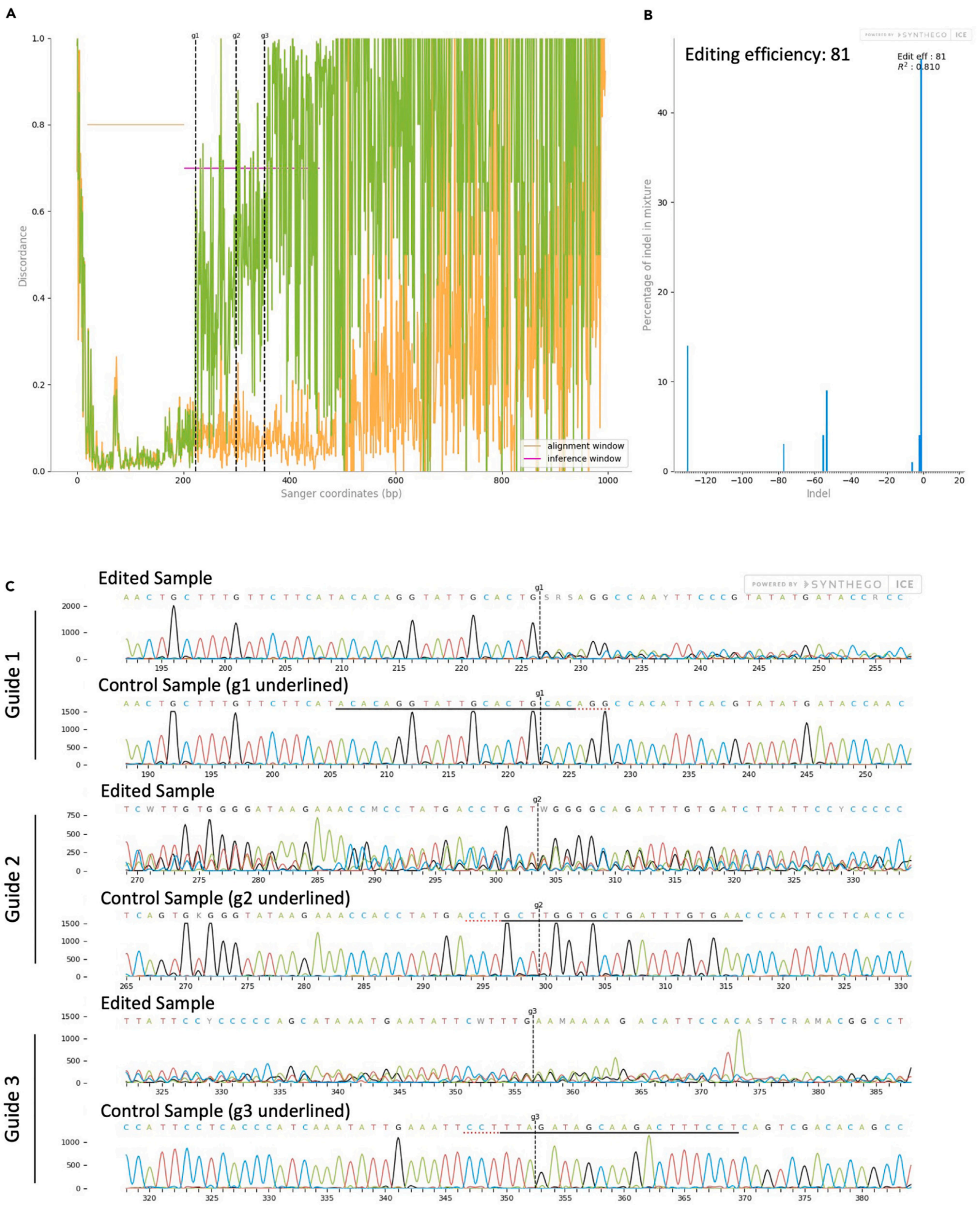
Analysis of Editing Efficiency of protein 1 in NK-92 cells as determined by Sanger sequencing and ICE analysis (A) An alignment plot displaying the aligned control (orange) and edited (green) sequences. (B) An indel plot depicting the anticipated range of insertions and deletions within the edited gene locus and the editing efficiency. (C) Traces of both edited and control DNA sequences. In the traces, the guide sequence is highlighted with a black underline, the PAM sequence is indicated in red, and the anticipated cleavage site is marked with a vertical dashed line.

**Figure 3:**
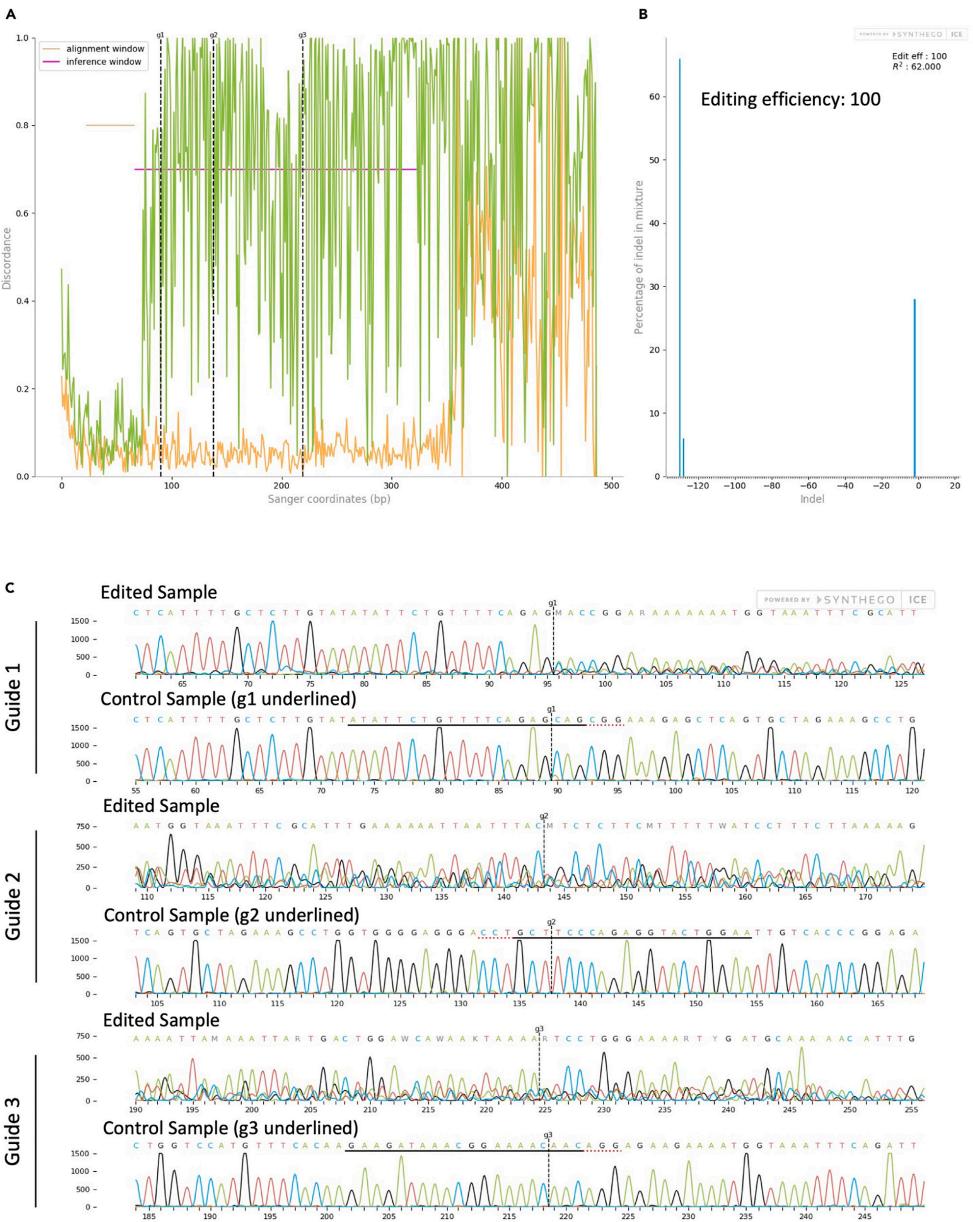
Analysis of Editing Efficiency of Protein 2 in human primary resting NK cells as determined by Sanger sequencing and ICE analysis (A) An alignment plot displaying the aligned control (orange) and edited (green) sequences. (B) An indel plot depicting the anticipated range of insertions and deletions within the edited gene locus and the editing efficiency. (C) Traces of both edited and control DNA sequences. In the traces, the guide sequence is highlighted with a black underline, the protospacer adjacent motif (PAM) is indicated in red, and the anticipated cleavage site is marked with a vertical dashed line.

***Note:*** The editing efficiency depicts the percentage of the cell population that has insertions or deletions. The Knockout Score indicates the proportion of cells harboring either a frameshift-inducing indel or a substantial indel within a protein-coding region, spanning 21 base pairs or more. These mutations are likely to result in a complete loss-of-function mutation. Finally, the R2 value (Pearson’s correlation coefficient) measures how well ICE's predicted indel distribution matches the Sanger sequence data from the edited sample.

66. After sequencing, use the .ab1 files from the control and edited samples to analyze InDel Percentages and Knockout scores using ICE analysis by Synthego.
  a. Label your sample in the ‘Label’ box.
  b. Paste your single/multi sgRNAs in the ‘guide sequence’ box.
  c. Do not fill in the ‘Donor sequence’, this is only used in case of CRISPR knockin.
  d. Drag and drop the .ab1 file of the control and experiment samples to the corresponding boxes.
  e. Analyze your samples. Figures 2 and 3 show representative results of the KO of protein 1 and 2 on genetic level in primary and NK-92 cells.

### Analysis of editing efficiency on protein level via flow cytometry

**Timing: 2–3 days**

Investigating both genetic and protein levels is important for comprehensive characterization of edited NK cells. While genetic analysis provides insights into the underlying genomic alterations induced by gene editing, protein-level assessment offers a measure of functional changes at the cellular level. To assess the edited NK cells on protein level, flow cytometry was used. Here, we explain how to stain for both intracellular and extracellular proteins. Representative results are shown below. Samples were acquired on the NovoCyte Quanteon Flow Cytometer System and analyzed using FlowJo (TreeStar). The following steps are applicable for both primary and NK-92 cells.

***Alternative:*** One can use alternative methods of choice to flow cytometry to determine the KO on protein level, such as Western blot or qPCR.

**CRITICAL:** Stain for both unedited and edited cells, as the unedited cells will show you the base level of your protein of interest and serve as control. Also include an FMO control, in which only the live/dead stain is used.

67. When targeting a cytoplasmic protein, cells need to be fixed and lysed to stain your protein of interest. We use the Foxp3 / Transcription Factor Staining Buffer Set (Thermo Fisher Scientific).
  ***Note:*** For nuclear proteins, please refer to the protocol of the vendor.
  ***Alternative:*** Depending on your intracellular target of interest, other kits may be preferred (e.g. Intracellular Fixation & Permeabilization Buffer Set, ThermoFisher).
  a. Take 200,000 electroporated cells and unedited cells and transfer to separate polystyrene FACS tubes.
  b. Prepare a single cell solution.
  c. Add 2 mL PBS-EDTA and centrifuge the cells at 450 × *g* for 5 min at 19°C–22°C. Decant the supernatant after centrifugation.
  d. Resuspend cells in 100 μL FACS buffer and stain the cell pellets with your fixable live/dead stain of choice.
  e. Incubate the cells for 15 min at 19°C–22°C in the dark.
  f. Wash the samples by adding 2 mL of PBS-EDTA and centrifuge at 450 × *g* for 5 min at 19°C–22°C. Discard the supernatant.
  g. Prepare a 1:4 dilution of Fixation/Permeabilization buffer; add 1 part Concentrate buffer to 3 parts Fix/Perm Diluent buffer.
  h. Fix the cells by adding 100 μL Fix/Perm buffer to each sample. Vortex samples.
  i. Incubate for 30 min at 19°C–22°C in the dark.
  j. Centrifuge at 600 × *g* for 5 min at 19°C–22°C. Discard the supernatant.
  k. Prepare a 1:10 dilution of the 10X Permeabilization solution in distilled water.
  l. Add 200 μL 1X Permeabilization solution per sample.
  m. Centrifuge at 600 × *g*, 19°C–22°C for 5 min. Discard supernatant.
  n. Resuspend the cell pellets in 100 μL 1X permeabilization buffer.
  o. Block with 2% human serum by adding 2 μL directly to the cells. Incubate at 19°C–22°C for 15 min.
  p. Without washing, add the recommended amount of conjugated antibody to the cells. Incubate for 45 min at 19°C–22°C. Protect from light.
  q. Add 200 μL 1X Permeabilization buffer and centrifuge at 600 × *g*, 19°C–22°C for 5 min. Discard supernatant.
  r. Resuspend stained cells in an appropriate volume of FACS Buffer.
  s. Analyze by flow cytometry. Figure 4 shows representative results for the knockout of cytoplasmic protein 2.

**Figure 4:**
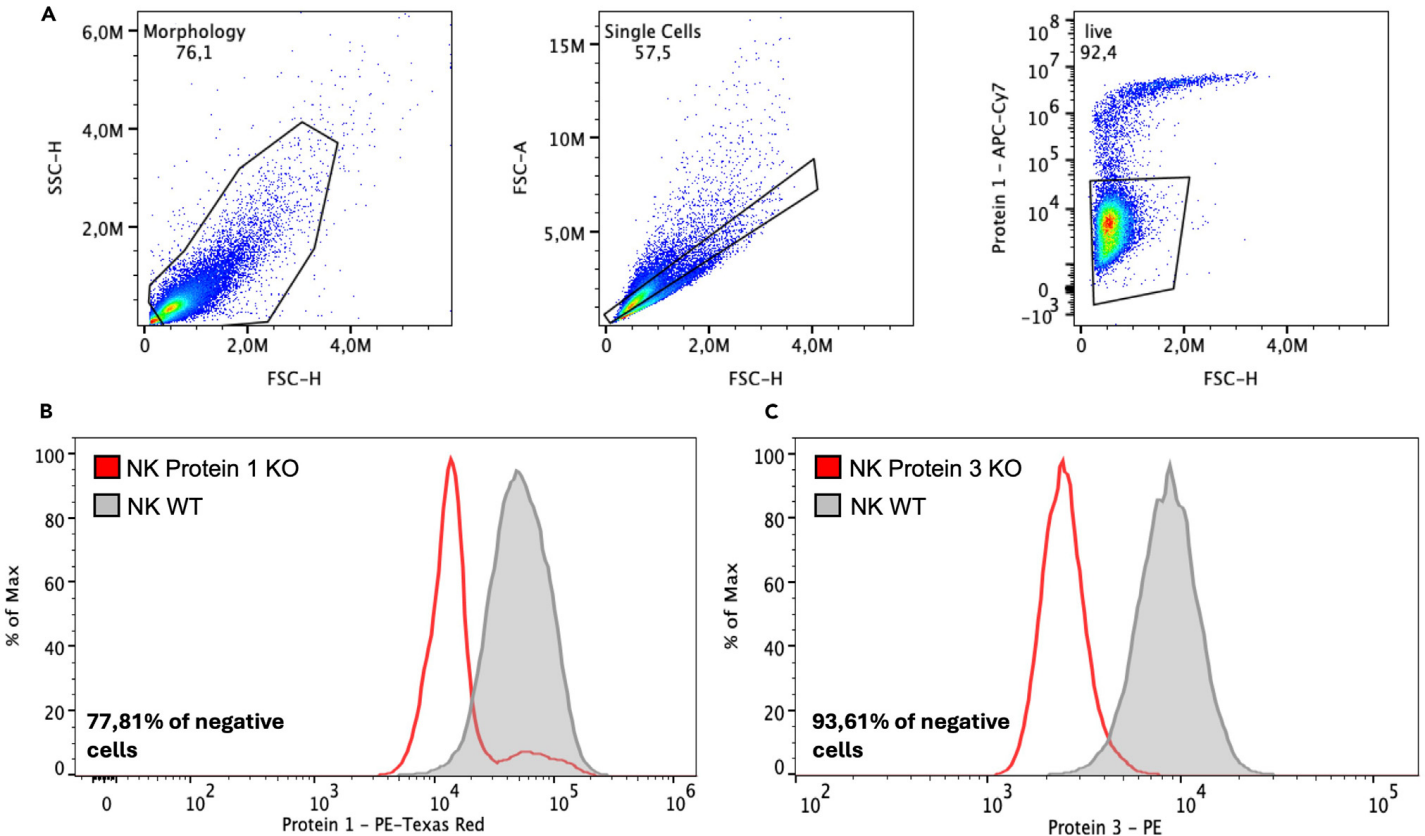
Representative flow cytometric plots of intracellular protein 1 and extracellular protein 3 in NK-92 NK cells (A) Gates were set on all cells gating on the forward scatter (FSC) and side scatter (SSC) pseudocolor dot plots. Next, single cells were selected. Live cells are defined as APC-Cy7 negative (Near Infrared Live/dead stain). (B) A histogram plot for intracellular protein 1 is shown for both wild-type (WT) and knockout (KO) cells. The % of negative cells was determined using FlowJo’s Overton Algorithm. (C) A histogram plot for extracellular protein 3 is shown for both wildtype (WT) and knockout (KO) cells. The % of negative cells was determined using FlowJo’s Overton Algorithm.
68. When targeting a protein that is expressed on the membrane of the cells, the following staining method can be used:
  a. Take 100,000 electroporated cells and unedited cells and transfer to separate polystyrene FACS tubes.
  b. Add 2 mL FACS buffer and centrifuge the cells at 450 × *g* for 5 min at 19°C–22°C. Decant the supernatant after centrifugation.
  c. Resuspend cells in 100 μL FACS buffer and stain the cell pellets with 1 μL fixable live/dead stain and 5 μL of the antibody of choice.
  d. Incubate the cells for 15 min at 19°C–22°C in the dark.
  e. Wash the samples by adding 2 mL of FACS buffer and centrifuge at 450 × *g* for 5 min at 19°C–22°C. Discard the supernatant.
  f. Acquire the stained cells on a flow cytometer. Figure 4 shows representative results for the knockout of extracellular protein 3.

**CRITICAL:** While this protocol provides initial recommendations for the concentrations of live/dead stain and antibodies, it is essential for researchers to adjust these levels through titration for their specific application.

## Expected outcomes

CRISPR-Cas9 edited human primary resting NK cells and NK-92 cells can be passaged every 3–4 days. Editing efficiency on genomic level can be determined earliest 24h after Nucleofection. Editing efficiency on protein level can be determined on a later time point, dependent on the turnover rate of the protein (see problem 5). Overall, our optimized protocol demonstrates high editing efficiencies can be obtained for resting human primary NK cells and NK-92 cells, as evidenced by our examples (81% and 100% according to ICE analysis, respectively). Still, editing efficiencies are dependent on several factors, including target gene of interest and sgRNA selection.^5^

## Limitations

This protocol is designed for cell quantities ranging from 1 × 10^6^ to 5 × 10^6^ NK cells per Nucleofection. It is worth mentioning that both cell viability and Cas9 uptake can be affected by the number of cells undergoing Nucleofection. The protocol may need adjustments to accommodate lower or higher cell numbers. It is also important to note that the Nucleofection programs in this protocol are optimized to achieve high editing efficiency while preserving average viability. However, it is essential to acknowledge the inherent trade-off between viability and efficiency in the nucleofector programs, a compromise we are unable to circumvent. While the transient expression of Cas9 protein ensures lower off-target editing, some genes might benefit from constitutive expression of Cas9.^6^ As discussed in the introduction, this can be achieved by viral transduction. However, this is predominantly applicable for NK-92 cells, which are easier to transfect than primary NK cells and comes at the expense of increased off-target risks.

## Troubleshooting

### Problem 1: Loss of viable cells after Nucleofection

A loss of viable cells is possible after Nucleofection (step 48). To enhance viability, consider implementing the following tips.

### Potential solution

To enhance viability, being well prepared is crucial. Streamline the process to reduce the time cells spend suspended in the P3 solution, emphasizing efficiency and swift execution. Handle cells post-Nucleofection with care to prevent additional stress. Utilize pre-warmed media for gentle resuspension and transfer of cells, ensuring minimal time outside the incubator to further support cell health. If your experimental set-up allows for cytokine stimulation, addition of (low) concentrations of cytokines (IL-15 and/or IL-2) to primary NK cells will enhance survival after Nucleofection.

### Problem 2: The concentration of amplified DNA is too low to sequence

When the concentration of amplified DNA is too low to Sanger sequence your samples after PCR (step 61), you might consider following solutions.

### Potential solution

Optimize PCR conditions to increase the yield of the amplified DNA. This includes adjusting primer concentrations, annealing temperatures, and extension times. Use a high-fidelity DNA polymerase to minimize errors and improve yield. You can perform the amplification in duplicate and pool the samples before PCR clean-up to increase the yield.

In the context of genomic DNA extraction, it is essential to use the appropriate amount of QuickExtract (QE) solution. Insufficient quantities may result in inadequate cell lysis, while excessive volumes can lead to genomic DNA dilution. If the isolated genomic DNA exhibits a sticky and slimy consistency after PCR, increasing the volume of QE solution is advisable. If you start with 0.5 million NK cells in 50 μL of QuickExtract (QE) solution and encounter a sticky result, you can attempt to resolve the pellet by increasing the volume of the solution to 100 μL.

### Problem 3: The sequencing quality is too low to use ICE analysis

An error message indicating that the quality of your input sequencing files is insufficient for ICE analysis can appear (step 66). Either the experiment files and/or the control files fail to meet the tool’s criteria for trace quality.

### Potential solution

Several factors could contribute to this issue.

• The PCR amplification is non-specific.

Ensure the PCR generates a single amplicon of the anticipated size by conducting a gel electrophoresis analysis prior to submitting your sample for sequencing. Design new primers or optimize the PCR melting and annealing temperatures.

• The Forward/Reverse primers are designed too close to the guide RNA target sequence(s).

For optimal ICE analysis, it is recommended to target a PCR amplicon length between 400 and 800 base pairs. When designing your primers, aim to maintain a minimum distance of 150 base pairs from the nearest sgRNA cut site to ensure effective sequencing coverage across the edit.

• The Sanger sequence trace is noisy, hindering reliable base calling by the provider.

Verify the quality of your sequencing trace for the edited sample by examining the region before the guide sequence. Noisy data is often characterized by noticeable secondary trace peaks. If the trace exhibits excessive noise, PCR cleanup should be performed. In addition, consider utilizing an internal nested primer for your sequencing to induce specificity and reduce background noise. Should noise issues persist, you might want to explore alternative Sanger sequencing providers. Finally, ensure that the “.ab1” files includes the quality score field; if it is missing, get in touch with your sequencing provider to request the inclusion of this information.

### Problem 4: Sequencing results indicate little to no editing efficiency

When ICE analysis indicates very low editing efficiencies, several adaptations might enhance the KO scores.

### Potential solutions

• Optimize Work Conditions

Perform tasks rapidly and under RNase-free conditions to maintain the integrity of the sgRNAs. Keep the guides on ice, wash cells with PBS, and use an RNase inhibitor to prevent RNA degradation. The use of an electroporation enhancer (e.g., Alt-R Cas9 Electroporation Enhancer, IDT) can also help to improve electroporation efficiency.

• Enhance Guide RNA Quality

Screen multiple guide RNAs for each targeted gene to identify the most effective ones. Consider employing a multi-guide approach to enhance knockout (KO) efficiency scores.

• Diversity in Donor Testing (for Primary NK Cells).

If working with primary NK cells, editing efficiencies may vary between donors. Test guide RNAs on cells from multiple donors to account for donor-specific effects. Include a minimum of three donors per guide RNA to assess variability.

• Enrichment of KO cells

If your research setup allows for single-cell sorting, this strategy could be used to enrich your KO population. This can be done using fluorescence-activated cell sorting (FACS) on a flow cytometer or magnetic-activated cell sorting (MACS) using a magnet as described for NK cell isolation in this protocol. This option will be mainly applicable for membrane proteins.

### Problem 5: Protein analysis indicates little to no editing efficiency

When encountering inconsistencies between knockout levels at the genetic and protein levels (steps 67–68), the following approach may offer a resolution.

### Potential solutions

Upon effective CRISPR-Cas9 gene editing, NK cells are not expected to generate new full-length proteins. Nevertheless, pre-existing proteins will continue to be expressed until they degrade upon reaching the end of their turnover period. To account for this, it is advisable to conduct a kinetic experiment measuring expression, spanning multiple days after CRISPR-Cas9 gene editing. This approach ensures a comprehensive understanding of protein dynamics post-editing.

## Resource availability

### Lead contact

Further information and requests for resources and reagents should be directed to and will be fulfilled by the lead contact, Jorrit De Waele (Jorrit.DeWaele@uantwerpen.be), upon reasonable request.

### Technical contact

Further information and requests for technical details should be directed to and will be fulfilled by the technical contact, Tias Verhezen (tias.verhezen@uantwerpen.be), upon reasonable request.

### Materials availability

This study did not generate new unique reagents.

### Data and code availability

This study did not generate datasets/code.
